# Supplementary material for: Systematic Characterization of GATA Transcription Factors in Liriodendron chinense and Functional Validation in Abiotic Stresses
Source: Plants (Basel). 2023 Jun 16;12(12):2349. doi: 10.3390/plants12122349 (PMC10302256; doi:10.3390/plants12122349)
Supplement: Supplementary file 1 [file plants-12-02349-s001.zip › Supplementary Figure .pdf]

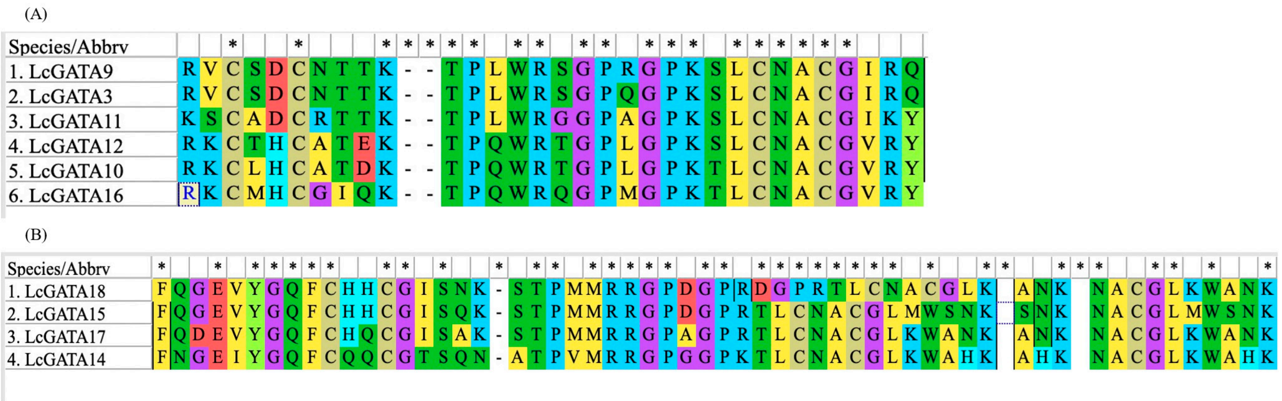

Supplementary Figure S1. LcGATA domain alignment from different groups.

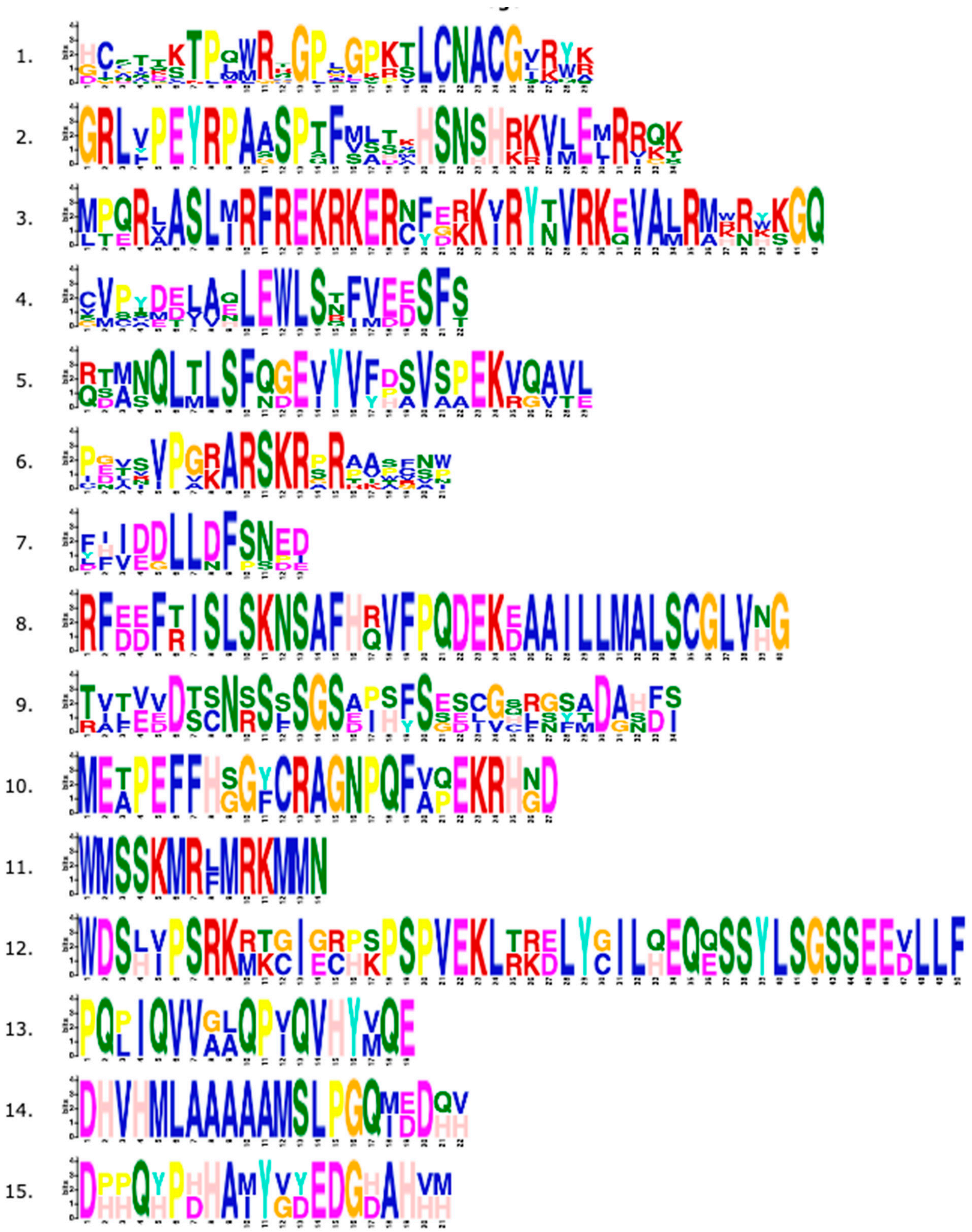

Supplementary Figure S2. LcGATA protein motif structure and arrangement, analyzed using the MEME online tool. The motifs detected were numbered 1–15.

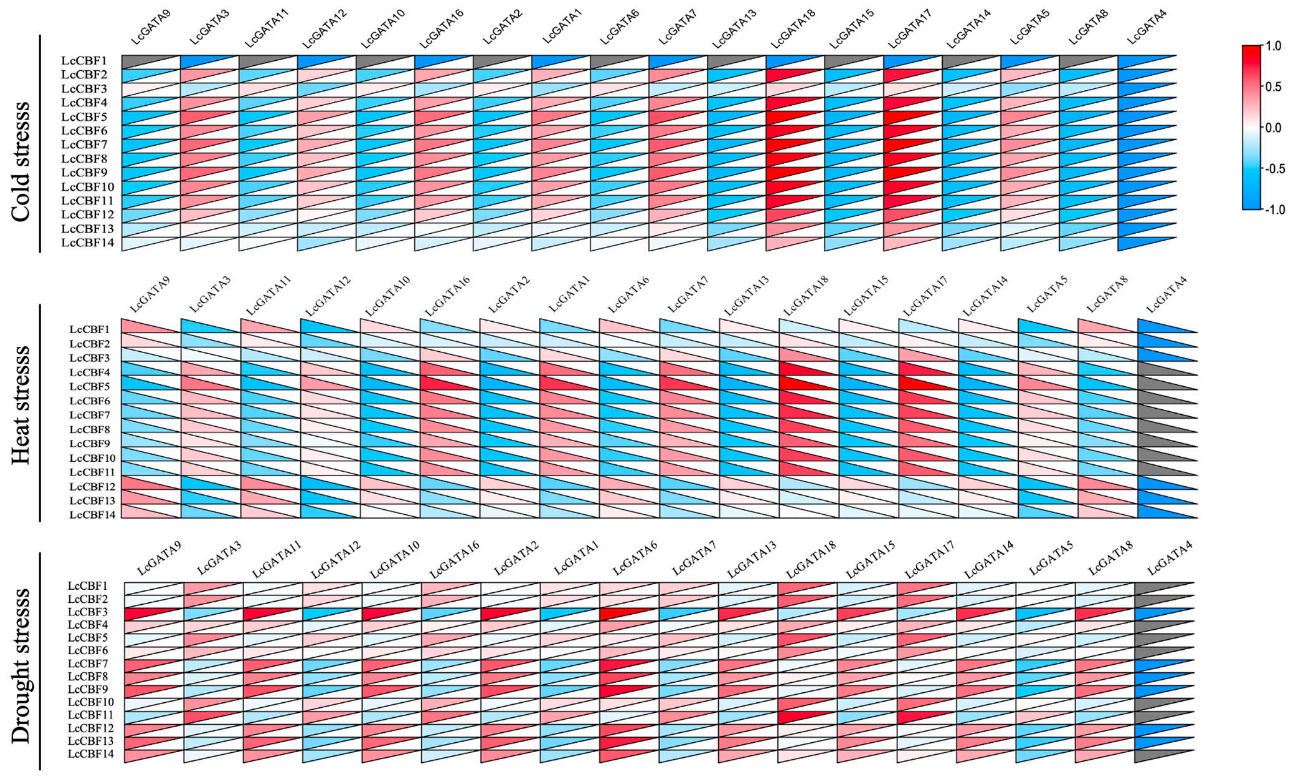

Supplementary Figure S3. Correlation analysis of the transcriptomic expression analysis between CBF and GATA genes in *L. chinense.*
