# Supplementary material for: Systematic Characterization of GATA Transcription Factors in Liriodendron chinense and Functional Validation in Abiotic Stresses
Source: Plants (Basel). 2023 Jun 16;12(12):2349. doi: 10.3390/plants12122349 (PMC10302256; doi:10.3390/plants12122349)
Supplement: Supplementary file 1 [file plants-12-02349-s001.zip › Supplementary Table .pdf]

Supplementary Table S1. qPCR primers used for LcGATA genes.

GENE ID	GENE NAME	DIR.	PRIMER
Lchi00761	LcGATA3	FOR	CAACAGCTGCAGCAGCTACGGCAA
Lchi00761	LcGATA3	REV	AACCATCACTCCACCATCTGCTGC
Lchi05395	LcGATA7	FOR	TTCGATGCCTCTTGATCCCCCA
Lchi05395	LcGATA7	REV	CCTTCTTGTGGGAATTCGAGTGCGCCG
Lchi03424.	LcGATA9	FOR	CTCTGAGAAATCTCAGCCATTTGATGATG
Lchi03424.	LcGATA9	REV	TGGCAGCCGCCATTGCCCTTCTTGCTTTC
Lchi08934	LcGATA10	FOR	GATTTCTTGCCGGAATCCAACGATGCGT
Lchi08934.	LcGATA10	REV	GCCCAACCTTCATGCTGACAAAGCATTC
Lchi01461.	LcGATA12	FOR	GTCGCGCAGTGAGTGCATTTCCTAACCC
Lchi01461.	LcGATA12	REV	GCACTCGAACTCTCACAGGAAGGTGATGGA
Lchi13044.	LcGATA14	FOR	CACCTCCACCGACCCGGTATCACGAAGATA
Lchi13044.	LcGATA14	REV	GCCTCTCCCGATCCCTTCTATGTCAGT
Lchi03334.	LcGATA15	FOR	TTCGTGGCGGAGTTTCAGTACGTGG
Lchi03334.	LcGATA15	REV	TGAGGGACCTGACAAGGAGCCTACCAG
Lchi17957.	LcGATA17	FOR	TTGAACGCCGTGGGAAGCCACCAAC
Lchi17957.	LcGATA17	REV	GAAGGAGTGTCGGCTTCCTCAGGCTTGGA
Lchi05152.	LcGATA18	FOR	CAGGTGTCTTTGATGAGGTGAACTGACCCT
Lchi05152.	LcGATA18	REV	GGATGCCATGGAAGCTGACCCGGAA
